# New evidence of predation on humans by cookiecutter sharks in Kauai, Hawaii

**DOI:** 10.1007/s00414-018-1786-8

**Published:** 2018-02-14

**Authors:** Agathe Ribéreau-Gayon, David O. Carter, Stephanie Regan

**Affiliations:** 1 Institute of Archaeology, University College London, 31-34 Gordon Square, London, WC1H 0PY UK; 2 Department of Security and Crime Science—Centre for the Forensic Sciences, University College London, 35 Tavistock Square, London, WC1H 9EZ UK; 3 Centre for the Forensic Sciences, University College London, 35 Tavistock Square, London, WC1H 9EZ UK; 4 Laboratory of Forensic Taphonomy, Forensic Sciences Unit, Division of Natural Sciences and Mathematics, Chaminade University of Honolulu, Honolulu, HI USA; 5 Kauai Police Department, Lihue, HI USA

**Keywords:** Scavenging, Cookiecutter shark, Bitemark, Shark attack, Taphonomy, Postmortem examination

## Abstract

The feeding patterns of species of large sharks on human corpses are well documented in the literature however, that of smaller sharks are less known. This may introduce uncertainty in the medicolegal conclusions. For that reason, accurate identification of patterns of shark predation is very relevant, specifically in areas bordered by the sea. In the case described here, an unidentified lesion was noted on the body of a victim of a scuba diving accident off the island of Kauai, in Hawaii. The aim of this study was to identify the origin of the lesion and investigate its potential to inform on the context of death and/or decomposition. The original outline of the lesion was digitally reconstructed to enable the collection of measurements which were compared with the literature and interpreted with an interdisciplinary approach. This approach permitted to determine that the macroscopic appearance and dimensions of the lesion (major axis = 3.53 cm) were consistent with a bitemark of a cookiecutter shark (*Isistius brasiliensis*). It was further determined that the bitemark was incomplete and that the specimen involved had a total length of about 24 cm and was likely to be a juvenile. This is the second report in the published literature of cookiecutter bitemarks on humans in the Hawaiian waters. This study brings new evidence-based insights into the interactions between cookiecutter sharks and human remains in marine environments and provides a valuable contribution to the knowledge base on the topic.

## Introduction

Water-related fatalities, whether accidental or criminal, have been a public concern for many years. For instance, drowning is the third cause of unintentional death in the world, causing about 360,000 fatalities per year [1], and an increasing number of displaced people have died at sea in the past few years (e.g. about 5000 deaths in the Mediterranean Sea only in the year 2016) [2, 3]. Because aquatic environments represent areas where people can lose their lives, the discovery of human remains in aquatic environments raises several forensic issues related to the aquatic context. Despite the high numbers of deaths in aquatic environments, the approaches available to establish postmortem conclusions of immersed remains, such as the postmortem submersion interval (PMSI) and the cause and manner of death, do not reach high levels of accuracy and objectivity [4–10]. Accurate identification of trauma is essential to establish the cause and manner of death. However, the identification of trauma, including distinguishing perimortem trauma (e.g. accidental or criminal) from postmortem trauma (e.g. animal scavenging or tidal action), on immersed corpses [11–13] is particularly challenging because of the lack of suitable methods. This may introduce uncertainty in the medicolegal diagnostic and the presentation of the evidence to Court.

Shark trauma is frequently encountered in marine environments around the world and causes a number of fatalities yearly [14]. In 2016, 126 shark attacks were reported worldwide, 11 of which occurred in Hawaii but none of which were fatal and three did not involve any injury at all (e.g. damage to equipment) [15]. Hawaii is the second most affected state in the USA with 153 shark attacks reported since 1837, far behind Florida which accounts for 778 attacks [16]. Interactions between sharks and humans, alive or dead, in Hawaiian waters are well documented and include the white shark (*Carcharodon carcharias* [Linnaeus, 1758]), tiger shark (*Galeocerdo cuvier* [Péron and Lesueur, 1822]), and grey reef shark (*Carcharhinus amblyrhynchos* [Bleeker, 1856]) [15, 16]. However, far less is known about the feeding patterns of smaller species of sharks, such as the cookiecutter shark (*Isistius* spp.), that inhabit these environments [9]. This is problematic for trauma identification in areas such as Hawaii. In addition to this gap in the knowledge, it should be noted that shark bites, just as any trauma, can be modified or even concealed because immersion in seawater can erode the edges of the bitemark, lead to the loss of body parts and accelerate the decomposition process [9, 14].

This paper presents the case of the body of a male diver who was recovered from the sea off the island of Kauai, Hawaii (USA). The body showed an unusual superficial C-shaped lesion on the thorax that was not specifically identified at the postmortem examination. The aim of the present paper is to retrospectively identify the origin of the lesion in views of demonstrating the importance of accurate trauma analysis in medicolegal death investigations in marine environments.

## Materials and method

### Background to the case

**Fig. 1:**
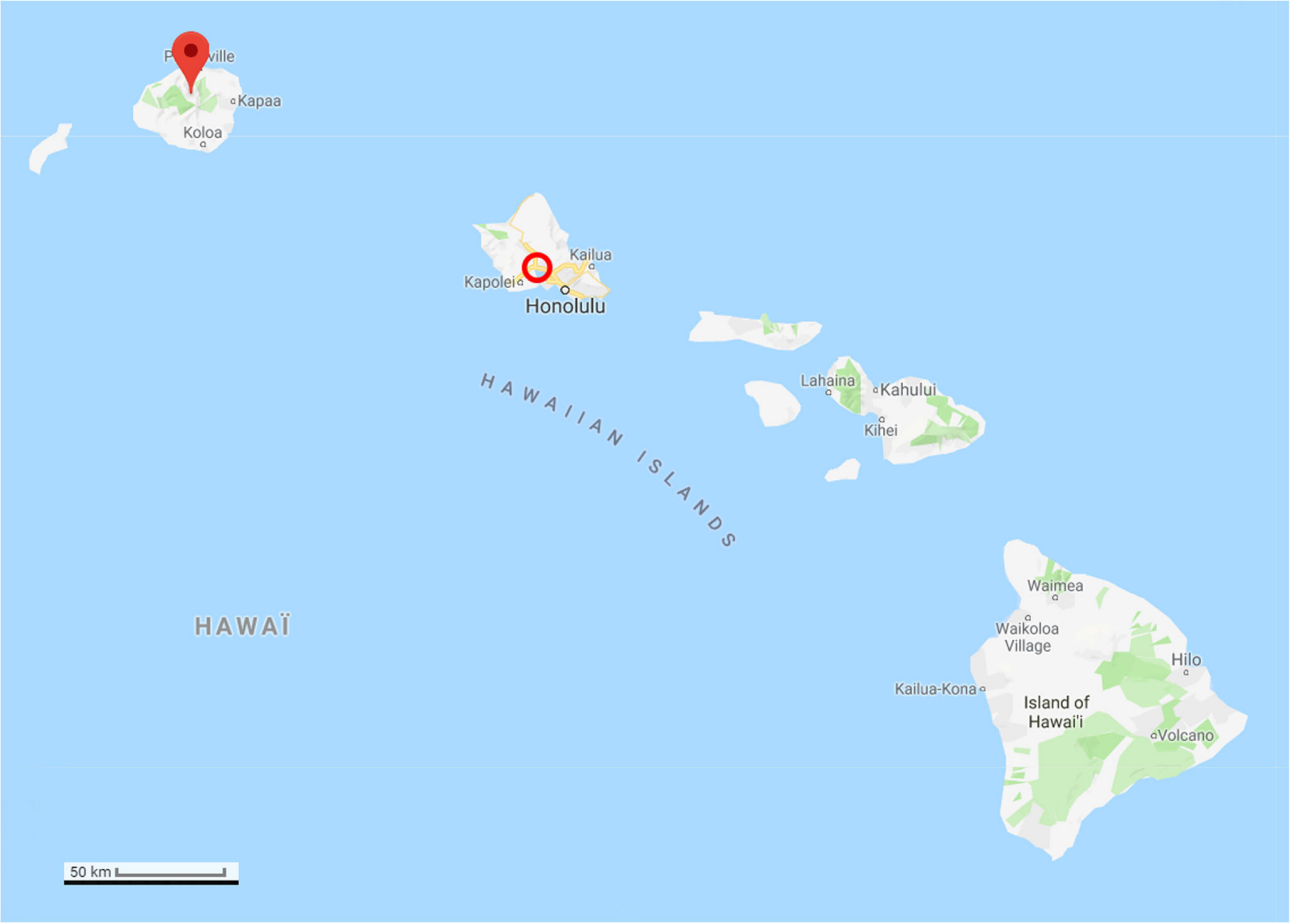
Map of the archipelago of Hawaii (USA), Google. (n.d.). (Google Maps, map of Hawaii, USA). Retrieved December 6, 2017, from https://goo.gl/Q5Rusg). The diver went missing in the island of O’ahu (circle), and his body was recovered in Kauai (pin)

**Fig. 2:**
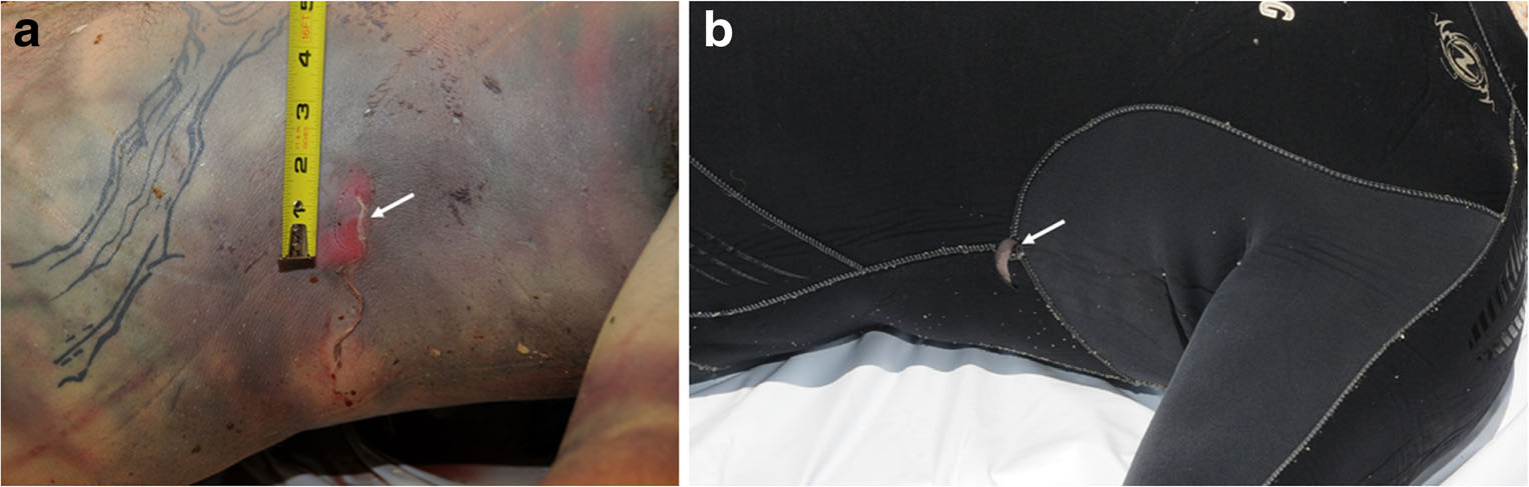
The victim at two different time points; postmortem examination on 16 June 2015 and time of recovery in Kauai 4 days earlier, on 12 June 2015. **a** The left thorax of the victim showed a distinct C-shaped lesion (arrow) that was associated with **b** a small C-shaped defect in the victim’s diving gear (arrow)

A 52-year-old male went missing during a scuba dive off the west coast of O’ahu in Hawaii, in the North Pacific approximately 21° 27′ N, 158° 15′ W, on 8 June 2015 (Fig. 1). On 12 June 2015, the body of the diver was recovered on the water surface off the coast of Kauai, about 100 miles north-west of O’ahu, in the northern part of the Hawaiian archipelago (Fig. 1). The postmortem examination of the victim performed by the Kauai pathologist on 15 June 2015 estimated a postmortem submersion interval (PMSI) of 4 days (date of submersion/death: 8 June 2015; date of recovery: 12 June 2015) [17]. The cause of death was diagnosed as a scuba diving accident, but with the possibility of perimortem shark involvement unable to be excluded as extensive scavenging by large sharks was observed. The autopsy photographs also show an unusual superficial C-shaped lesion located on the left side of the thorax, the origin of which was not specifically identified in the autopsy report (Fig. 2a). The lesion was located in the sagittal plane (perpendicular to the body of the victim) with its tips oriented towards the head of the victim. A C-shaped defect of comparable size, shape, and location was also observed in the victim’s diving gear (Fig. 2b).

### Analytical protocol

**Fig. 3:**
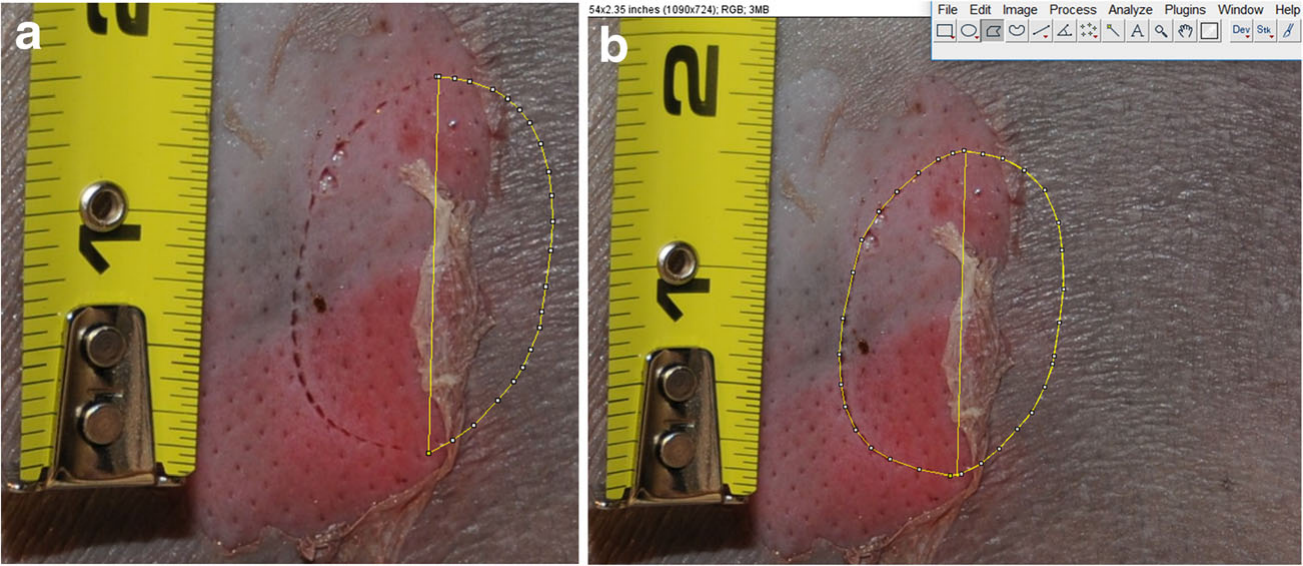
Ongoing measurement of the lesion with the programme ImageJ for PC. **a** The reconstruction of the outline of the bitemark enabled to **b** collect measurements from the reconstructed bitemark

A thorough retrospective analysis of all the sources available on the case was conducted (police reports, US Coast Guard reports, autopsy report, and digital photographs taken at recovery and postmortem examination). Data on the macroscopic characteristics and measurements of the lesion were collected from the photographs. The distance between the tips of the C-shaped lesion was used as a proxy for the major axis (or maximum length) of the lesion and was digitally measured with the programme ImageJ for PC [18]. The original outline of the lesion was then digitally reconstructed by mirroring the existing C-shaped lesion (Fig. 3). This procedure facilitated the collection of measurements on the perimeter, area, minor axis (or minimum length), as well as the major axis/minor axis ratio used as a proxy for shape of the lesion. Of the various measurements collected from the lesion, only the major axis was further investigated as it was the only measurement possible to take from the original data, and not from the reconstructed data, and it is a standard measurement in the literature on animal bitemarks [19–26]. The total length (TL) of the animal potentially involved in the lesion was calculated with the formula developed by Muñoz-Chápuli [22] which is based on the maximal length of the lesion that is used as a proxy for the width of the shark’s mouth. In views of identifying the origin of the lesion, the data collected were interpreted by expert forensic anthropologists and ichthyologists in consultation with medicolegal doctors before being rigorously compared with the published literature.

## Results

**Fig. 4:**
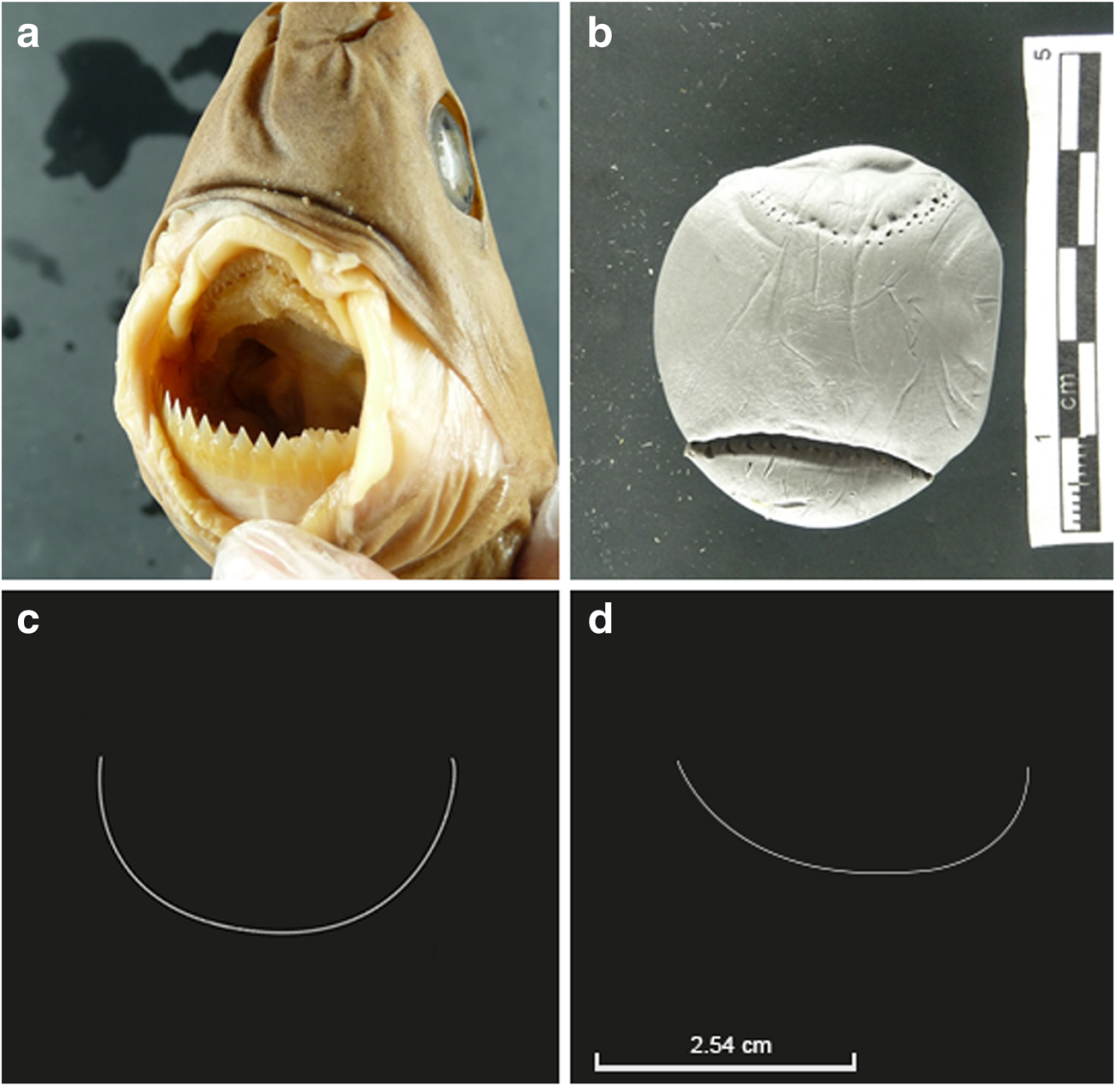
Specimen of cookiecutter sharks of the genus *Isistius brasiliensis* and the unsuccessful C-shaped bitemarks they can produce. **a** *Isistius brasiliensis* are small brownish cigar-shaped sharks sporting fleshy lips and extremely sharp teeth. This specimen from the Natural History Museum in Paris, France was used to **b** experimentally reconstruct a bitemark in plasticine. **c** The lesion analysed in the present case from Kauai, in Hawaii, shares some macroscopic similarities with **d** a superficial C-shaped lesion on the thorax of a male diver off to Maui, also in Hawaii, reported by Honebrink et al. [20]. The outline of the bitemark was reconstructed after Honebrink et al. (2001)

The arc of the lesion analysed in the present case was composed of a minimum of 22 incised line-shaped notches. This pattern was interpreted as tooth notches from an animal, possibly a marine animal. The lesion showed a major axis of 3.53 cm. Macroscopically, the lesion was found to be consistent with the activity of a marine animal. Furthermore, the bitemark was readily distinguishable from the large, deep, and V-shaped bitemarks observed on the corpse of the victim—including on the head, thorax and abdomen—that were likely caused by larger sharks, such as tiger sharks, whose bite patterns are well documented [11–13, 27–32]. It is thus likely that the present lesion corresponds to a bitemark from a species of small marine sharks. Precisely, the length of the major axis of the lesion fits well within the known range (1–10 cm) for bitemarks caused by *Isistius* spp. (the so-called cookiecutter sharks), two cigar-shaped species of dwarf sharks: *I. brasiliensis* (Quoy and Gaimard, 1824) and *Isistius plutodus* (Garrick and Springer, 1964) [9, 20, 22, 23, 26, 33]. Five cookiecutter bitemarks with a diameter of 3 cm were reported on a corpse recovered off O’ahu, Hawaii [26]. Furthermore, in the case of the 2009 Yemenia airplane crash in the Indian Ocean, 88 off the 560 (15.7%) cookiecutter bitemarks identified on the victims were encompassed within a range of 3–4 cm [9]. More specifically, the number of tooth notches visible on the lesion examined here (*n* ≥ 22) suggests the involvement of *I. brasiliensis*, a ≤ 56-cm species, whose lower jaw contains 25 teeth or more (Fig. 4a), contrary to that of *I. plutodus* whose lower jaw only contains a maximum of 19 teeth [34].

More precisely, the specimen involved in the present bitemark had an estimated total length (TL) of about 24 cm. The estimated TL of the shark in the present case is below the known range for mature *I. brasiliensis* specimens (13 to 36 cm for males and up to 39 cm for females [23] and thus indicates that the bitemark was likely caused by a juvenile individual. The TL is consistent with the average length of 24.29 cm calculated by Jahn and Haedrich [23] from 111 *I. brasiliensis* bitemarks on various fishes. Juvenile *I. brasiliensis*, just like adults, are known for preying on marine animals [22] and human cadavers [9]. The mark analysed further corresponds to the imprint of the lower jaw of the shark. This hypothesis is corroborated by the feeding behaviour of *I. brasiliensis* who first attach to its prey by incising the flesh with its sharp spear-headed lower teeth [35]. This hypothesis was experimentally investigated at Paris Natural History Museum where preserved specimens of *I. brasiliensis* were used to reproduce bitemarks in a piece of plasticine to analyse the imprint left by the jaws (Fig. 4b)*.* The lower jaw produced a deep serrated C-shaped incision that nearly detached a piece of plasticine (bottom of the image), while the upper jaw left a C-shaped series of superficial perforations (top of the image). This experiment confirmed that the bitemark analysed in the present case is consistent with a light pressure from the lower jaw of *I. brasiliensis*. The orientation of the bitemark further indicates that the shark was aligned parallel to the body of the victim (in the sagittal plane) when it bit, as the greatest axis of the bitemark, which corresponds to the shark’s mouth width, is perpendicular to the body (in the transverse plane) [23, 36].

The thickness of the diving gear of the victim is likely to have prevented the shark from completing its bite. Similar C-shaped defects caused by cookiecutter sharks to various fabrics, such as nets, have been reported [37]. Unsuccessful C-shaped cookiecutter bitemarks on humans have been reported twice in the published literature [20, 21], including one on the thorax [20]. In March 2009, a cookiecutter attack on a long-distance swimmer was reported at night time in the Alenuihaha Channel that links the main island of Hawaii to Maui, further south of Kauai (Fig. 1) [20]. The thorax of the swimmer exhibited a superficial C-shaped lesion (Fig. 4c), comparable to that analysed in the present case (Fig. 4d), approximately 5 cm in diameter. The lesion could have been the result of an aborted bite, as reported on marine mammals in biology literature [38]. The abortion of the attack may be explained by the thin density of flesh on the thorax, compared with fleshier areas on the body, such as the calves where the swimmer in question exhibited a complete deep cookiecutter bitemark [20]. A large-scale study conducted on the victims of the 2009 Yemenia airplane crash in the Indian Ocean identified the thorax area as the most frequently bitten by cookiecutter sharks (29.1% of the 560 bitemarks analysed) [9, 39]. As a complementary argument in favour of the involvement of *I. brasiliensis* in the bitemark analysed here, the species has been reported a few times in Hawaii, where the present case occurred [20, 33].

In conclusion, both the macroscopic characteristics of the lesion and the geographic location of the incident suggest that the lesion examined here was an unsuccessful bitemark, likely to have been caused by a juvenile specimen of *Isistius brasiliensis*. This diagnosis was confirmed by an expert ichthyologist who has been consulted on numerous cases of shark attacks across the world [40, 41]. This interdisciplinary approach proved successful in other cases of shark trauma on human remains [9, 14].

**Fig. 5:**
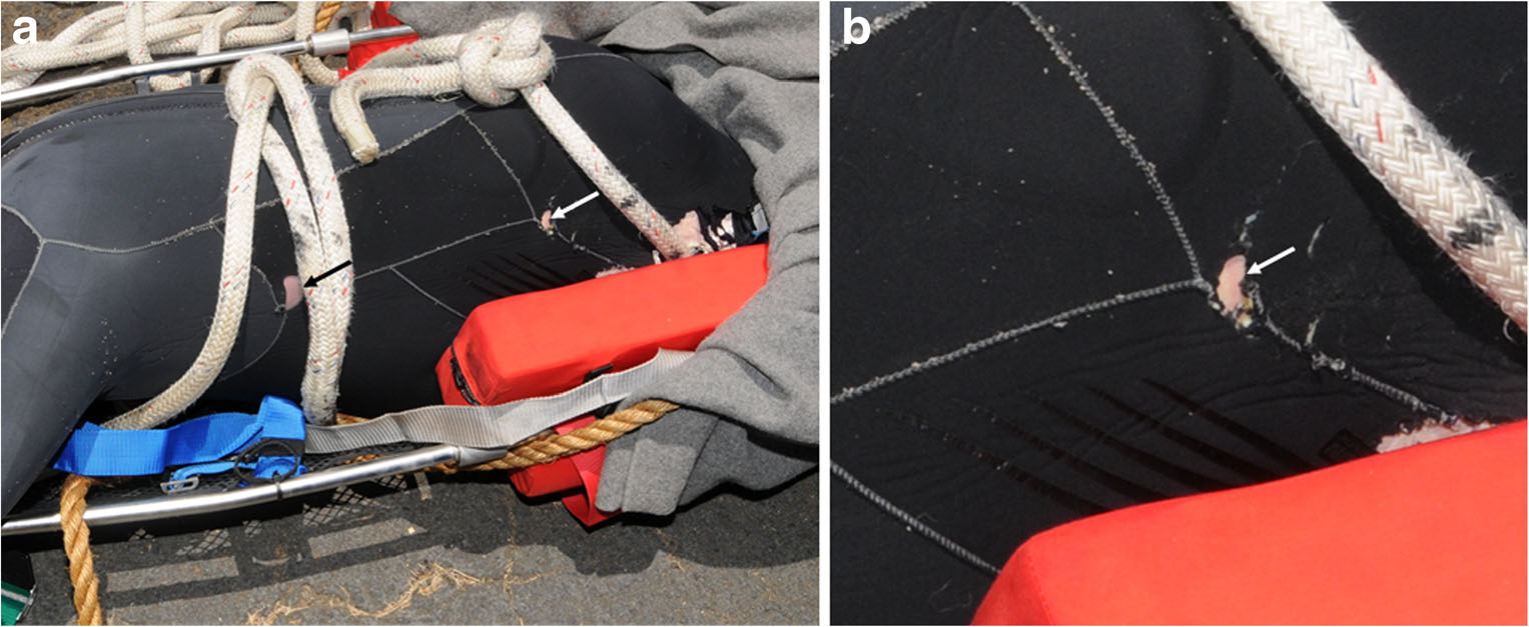
Two C-shaped defects in the victim’s diving gear at time of recovery in Kauai (Hawaii), 12 June 2015. **a** Close-up photograph showing a small C-shaped defect in the victim’s diving gear (white arrow), on the same side as the defect identified as a cookiecutter bitemark (black arrow). **b** Close-up photograph of the left hip of the victim showing a second small C-shaped defect in the victim’s diving gear (arrow)

The possibility that the corpse of the present victim actually sustained more than one cookiecutter bite cannot be ruled out for two reasons: (i) the photographs available did not enable to see all the surfaces of the body and (ii) some small bitemarks may have been concealed by larger shark bitemarks. For example, a second C-shaped defect is visible in the victim’s diving gear around the left hip area and could be indicative of another bite (Fig. 5a, b).

## Discussion

The present findings add to a small collection of evidence of postmortem scavenging by cookiecutter sharks on human corpses immersed in oceanic environments. The findings also confirm the existence of interactions between the cookiecutter sharks and human cadavers in the Hawaiian waters, as reports in the published literature were scarce. This provides valuable additional knowledge on the ecology of these sharks which is poorly understood to date [9]. Further study on the timing of feeding of cookiecutter sharks could prove valuable to refine PMSI estimates in future investigations of unwitnessed deaths in oceanic environments as robust and accurate methods are currently lacking [5, 7, 9].

The present conclusions are derived from a single bitemark and must therefore be interpreted and used with caution. However, these results contribute to build a broader understanding of human-cookiecutter shark interactions in oceanic environments and will thus feed an ongoing anonymised database of cookiecutter bitemarks on human corpses. These data will enable similar lesions to be diagnosed more accurately in the future and ultimately help to gain a better understanding of the range of expressions of cookiecutter bitemarks, including their macroscopic appearance and distribution patterns on the corpse. This background knowledge will provide means to evaluate the evidential value of cookiecutters’ signatures on human corpses, as it appears that the forensic relevance of cookiecutter sharks in oceanic environments has been underestimated in forensic investigations so far [9]. The present findings and the research avenues they open bring a valuable contribution to various disciplines, such aslegal medicine, forensic anthropology, and the law, but also marine biology and ecology.
